# Supplementary material for: TrkA undergoes a tetramer-to-dimer conversion to open TrkH which enables changes in membrane potential
Source: Nat Commun. 2020 Jan 28;11:547. doi: 10.1038/s41467-019-14240-9 (PMC6987127; doi:10.1038/s41467-019-14240-9)
Supplement: Supplementary file 1 — Supplementary Information [file 41467_2019_14240_MOESM1_ESM.pdf]

Supplementary Information

TrkA undergoes a tetramer-to-dimer conversion to open TrkH which enables changes in membrane potential

Zhang et al.

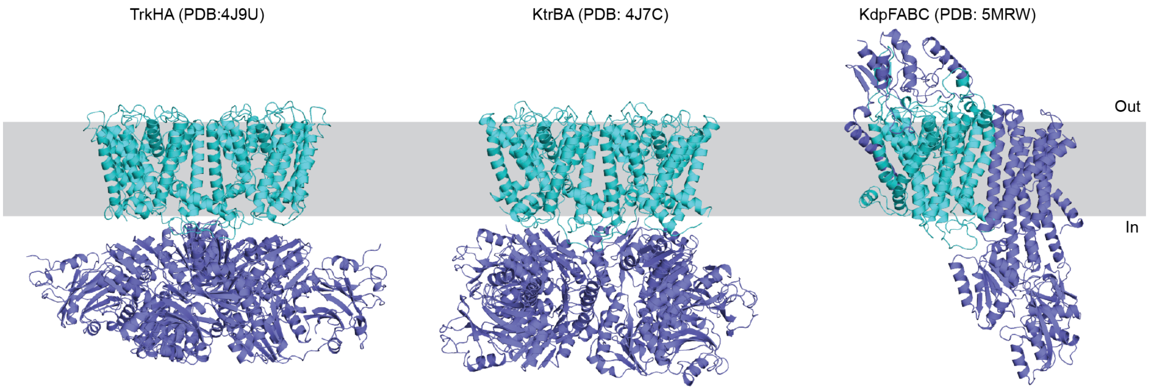

Supplementary Figure 1. Previously reported SKT structures

Structures of the TrkH-TrkA, KtrB-KtrA, and KdpFABC complexes rendered in cartoon and viewed within the plane of the membrane[1–3]. Cell membrane is shown as a grey box and the top is the periplasmic space.

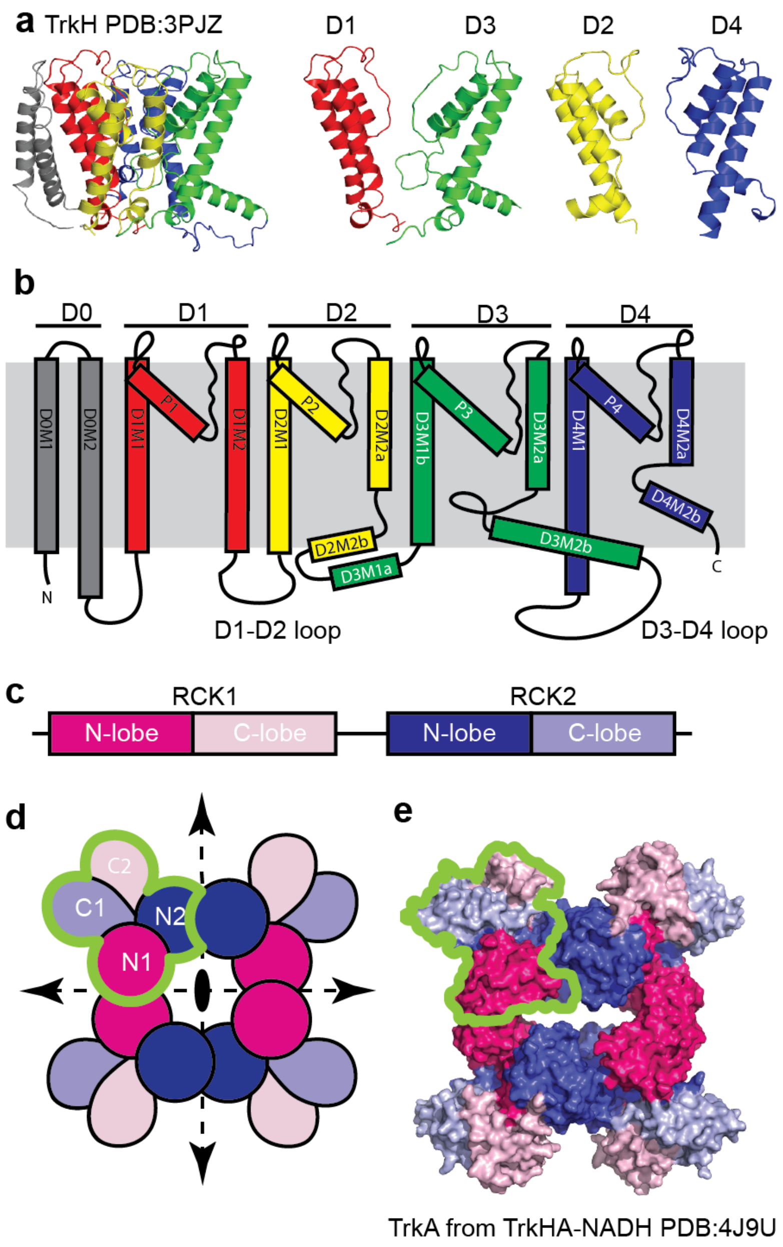

Supplementary Figure 2. Structure and assembly of TrkH and TrkA.

a. TrkH protomer (Left) rendered in cartoon with each M1-P-M2 motif in a different color. Diagonally opposing M1-P-M2 motifs, D1 and D3 (Middle) and D2 and D4 (Right), are shown for clarity. **b.** Topology diagram of a TrkH protomer shown with the periplasmic side up. **c.** Schematic illustration showing the domains within a TrkA protomer. **d.** Schematic illustration showing the assembly of TrkA tetramer, with a D_2 symmetry. The 2-fold axes are marked as either an oval dot or dashed lines with arrowheads. **e.** TrkA tetramer rendered in surface representation with each domain colored as in **c**. The green outline marks a single protomer.

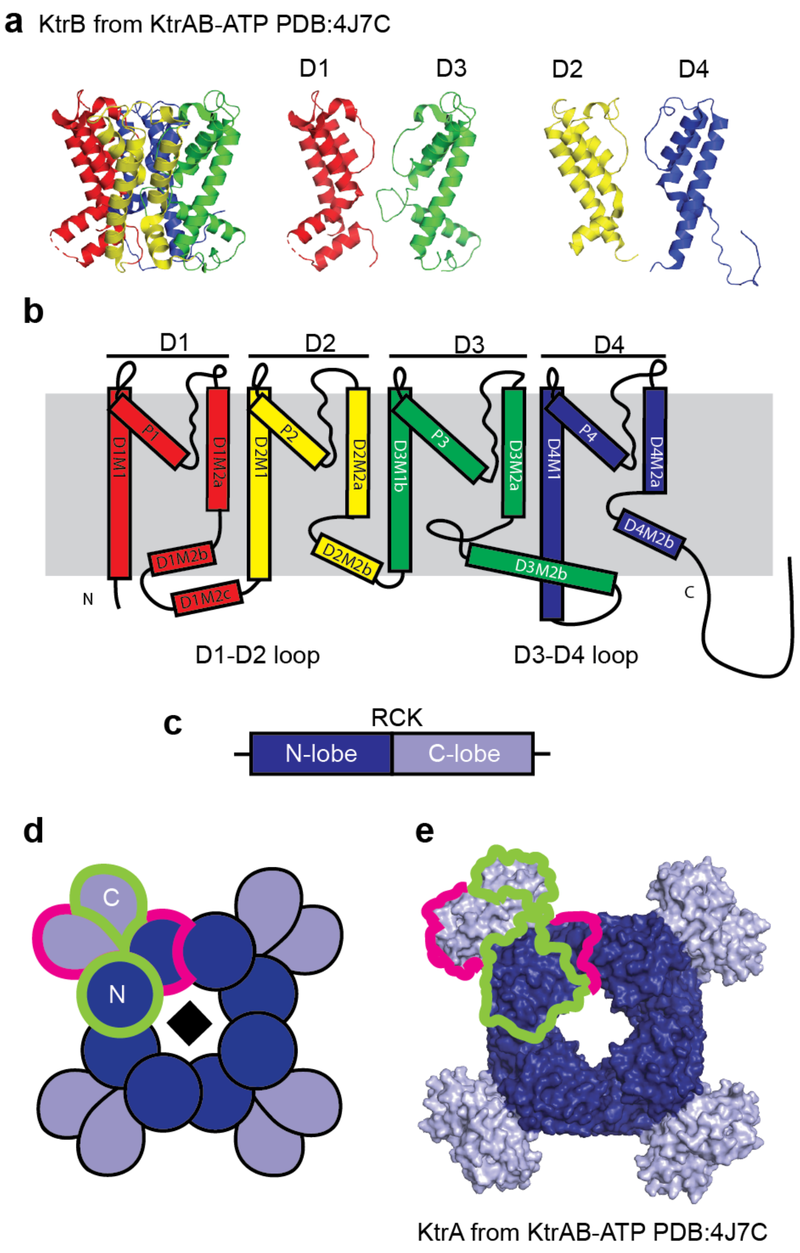

Supplementary Figure 3. Structure and assembly of KtrB and KtrA.

a. KtrB protomer (Left) rendered in cartoon with each M1-P-M2 motif in a different color. Diagonally opposing M1-P-M2 motifs, D1 and D3 (Middle) and D2 and D4 (Right) are shown for clarity. **b.** Topology diagram of a KtrB protomer shown with the periplasmic side up. **c.** Schematic illustration showing the domains within a KtrA protomer. **d.** Schematic illustration showing the assembly of KtrA octamer, with the 4-fold axis marked as a diamond. **e.** KtrA octamer rendered in surface representation with each domain colored as in **c**. The green and pink outline each marks a single protomer within a KtrB dimer.

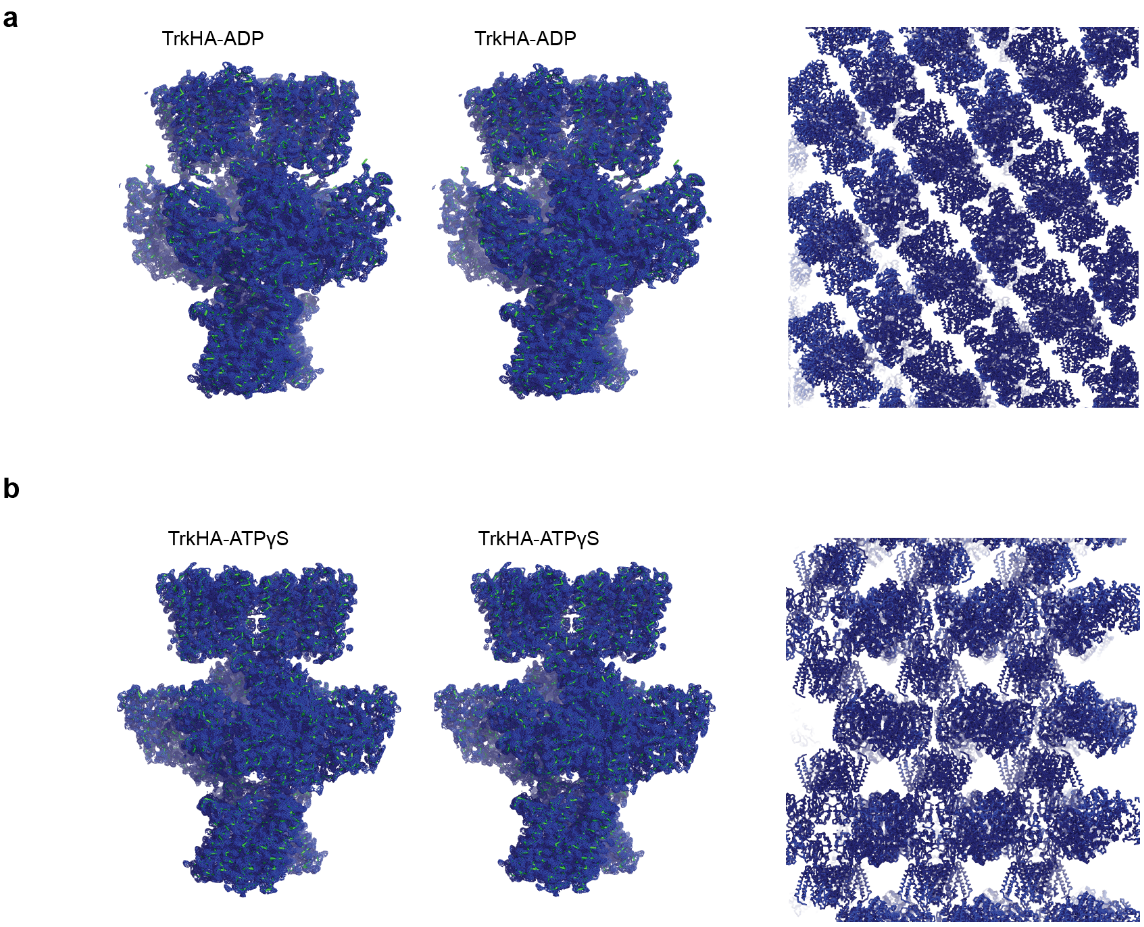

Supplementary Figure 4. Electron densities and crystal packing of TrkHA-ADP and TrkHA-ATPγS

a. Left, stereo image of 2Fo-Fc electron density map contoured at 1.0 σ (blue mesh) overlaid with a ribbon representation of TrkHA-ADP structure. Right, crystal packing of TrkHA-ADP. **b.** Left, stereo image of 2Fo-Fc electron density map contoured at 1.0 σ (blue mesh) overlaid with a ribbon representation of TrkHA-ATPγS structure. Right, crystal packing of TrkHA-ATPγS.

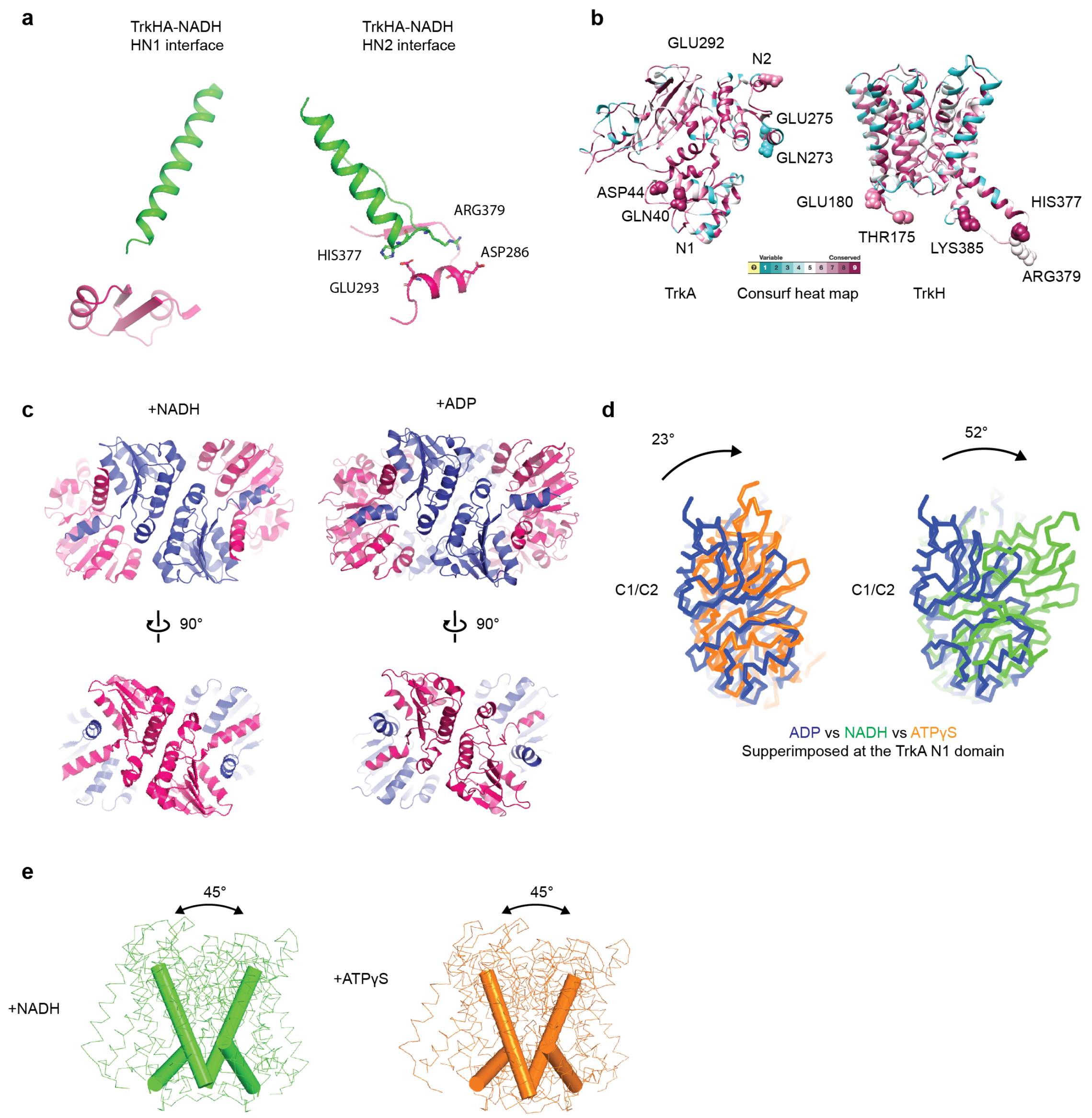

Supplementary Figure 5. Conformational changes in TrkHA

a. HN1 and HN2 interfaces from TrkHA-NADH. Fragments from TrkH rendered in cartoon are shown in green and fragments from TrkA rendered in cartoon are shown in hotpink. **b.** Consurf heat map of TrkH and TrkA from TrkHA-ADP rendered in cartoon. **c.** TrkA gating rings from TrkHA-NADH (Left) and TrkHA-ADP (Right) viewed from the N2-N2 (Top) and N1-N1 (Bottom) interfaces. **d.** Comparison of the relative positions of the C1/C2 domains. TrkA protomers rendered in ribbons are superimposed at the N1 domains. **e.** TrkH from TrkHA-NADH (Left) and TrkHA-ATPγS (Right) rendered in ribbons viewed within membrane. The D4M1 and D3M2b helices at the dimer interfaces are shown cylinders.

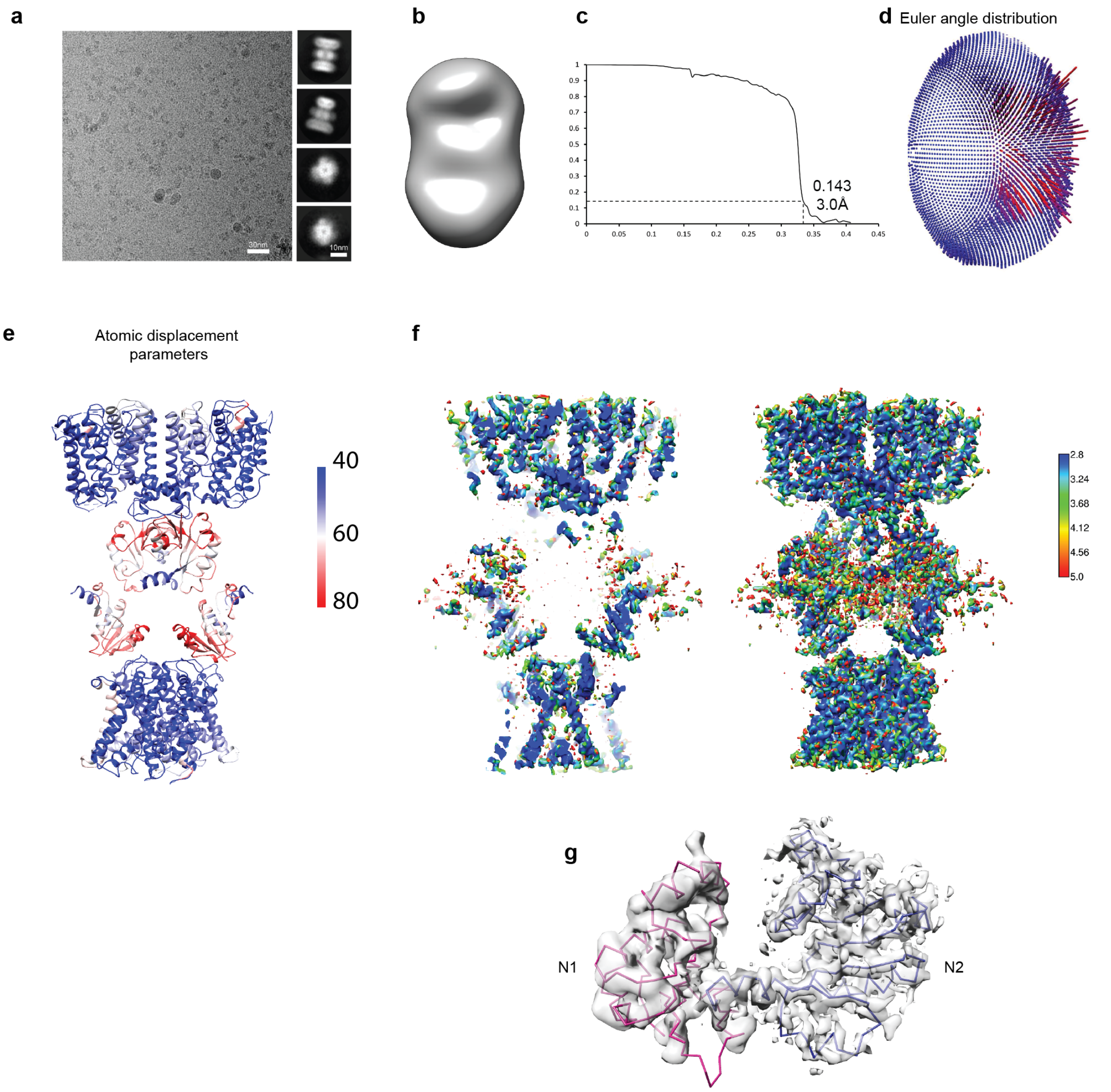

Supplementary Figure 6. Cryo-EM of TrkHA-ATP

a. Representative micrograph and selected 2d class averages of TrkHA-ATP. **b.** Initial model of TrkHA-ATP. **c.** Fourier shell correlation plot. **d.** Euler angle distribution of particles reconstituted into the final map. **e.** Atomic displacement parameters (also known as B-factors) of TrkHA-ATP[4]. **f.** Local resolution of TrkHA-ATP determined by Resmap[5]. **g.** Density map of TrkA from TrkHA-ATP shown in surface representation. N1 domain rendered in ribbons is colored in hot pink. N2 domain rendered in ribbons is colored in blue.

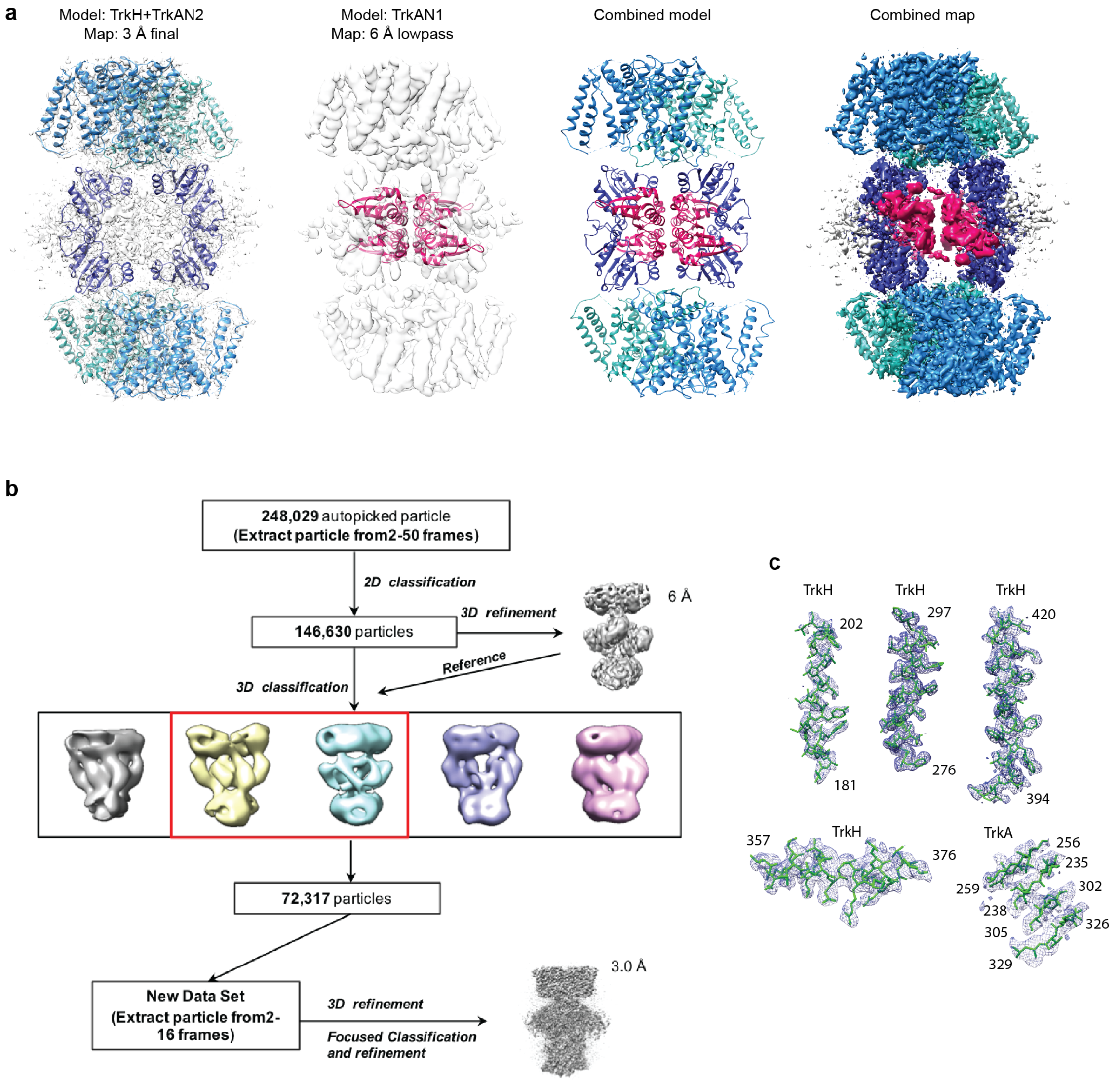

Supplementary Figure 7. Model building and data processing of TrkHA-ATP

a. Building and refining the structural model of TrkHA-ATP using a combination of the final 3 Å and an intermediate 6 Å low-pass filtered map. TrkH and the N2 domain of TrkA were build using the high resolution map. The N1 domain of TrkA was docked into the 6 Å low-pass filtered map guided by several of the helices and the general shape of the domain. The two models and maps were then combined. **b.** Workflow chat of data processing. **c.** Representative Cryo-EM densities of TrkHA-ATP. The residue numbers are labeled on the side (TrkH: 181-202, 276-297, 357-376, or 394-420; TrkA: 235-238, 256-259, 302-305, or 326-329)

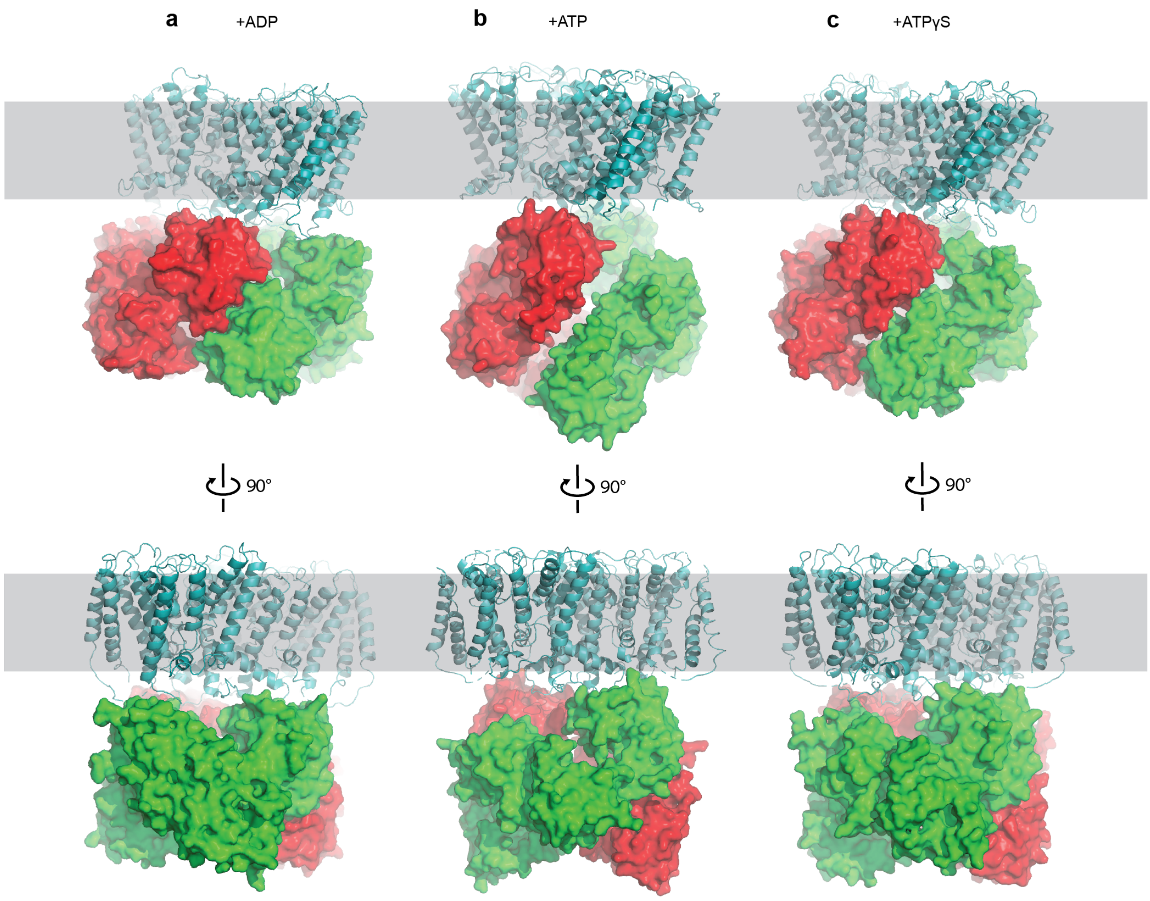

Supplementary Figure 8. Tetramer to dimer conversion of TrkA.

Structures of TrkHA-ADP (**a**), TrkHA-ATP (**b**), and TrkHA-ATPγS (**c**) with TrkH rendered in cartoon (teal) and TrkA rendered in surface viewed within the plane of the membrane in two directions. The TrkA protomers that belong to different TrkA dimers in TrkHA-ATP are colored in green and red respectively.

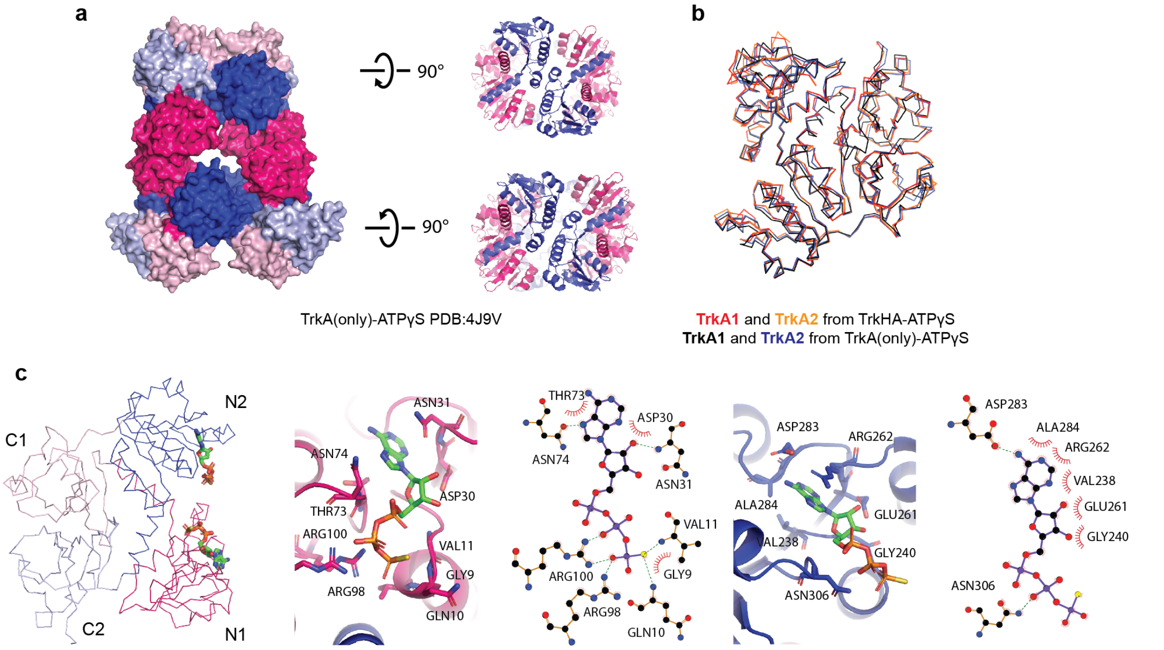

Supplementary Figure 9. Comparison of TrkA in isolation and in the TrkHA complex in the presence of ATPγS

a. Surface representation of TrkA gating ring from the structure of isolated TrkA-ATPγS (PDB: 4J9V). Two N2-N2 interfaces are displayed on the right. **b.** Superposition of TrkA protomers from TrkHA-ATPγS and TrkA-ATPγS (PDB: 4J9V). There are 2 TrkA proteomers (TrkA1 and TrkA2) in each unit cell from these structures. **c.** Isolated TrkA bound with ATPγS (PDB: 4J9V) rendered in ribbons and the zoom in views of N1 and N2 nucleotide binding interfaces. The nucleotide-protein interaction diagrams are drawn using LigPlot+[6].

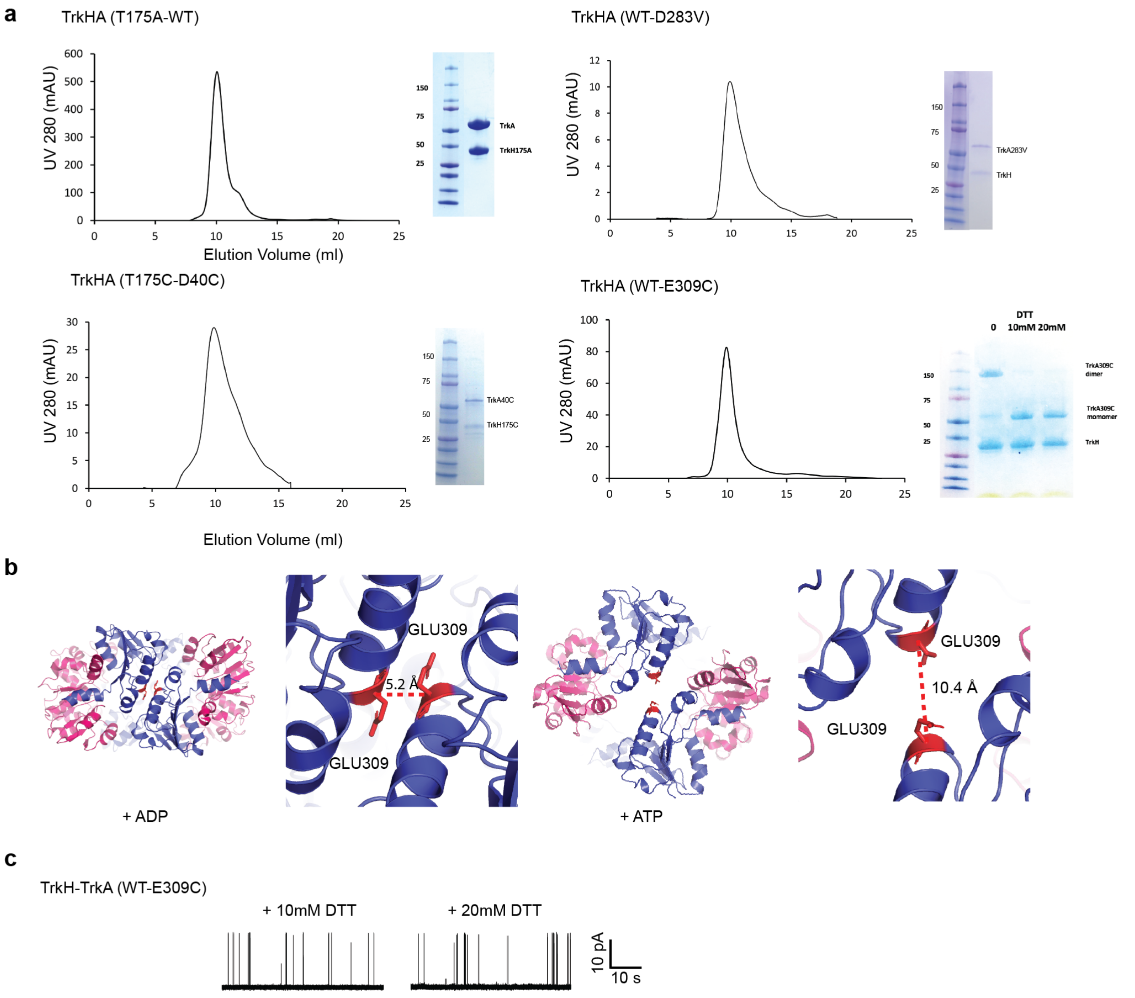

Supplementary Figure 10. Mutagenesis experiments

a. Size exclusion chromatogram and SDS-PAGE of different TrkHA mutants. **b.** Cartoon representations of N2-N2 interactions in TrkHA-ADP or TrkHA-ATP. The distance between Cαs from GLU309s are marked with the red dashed lines. **c.** Current traces of TrkHA (wt-E309C) in the presence of DTT.

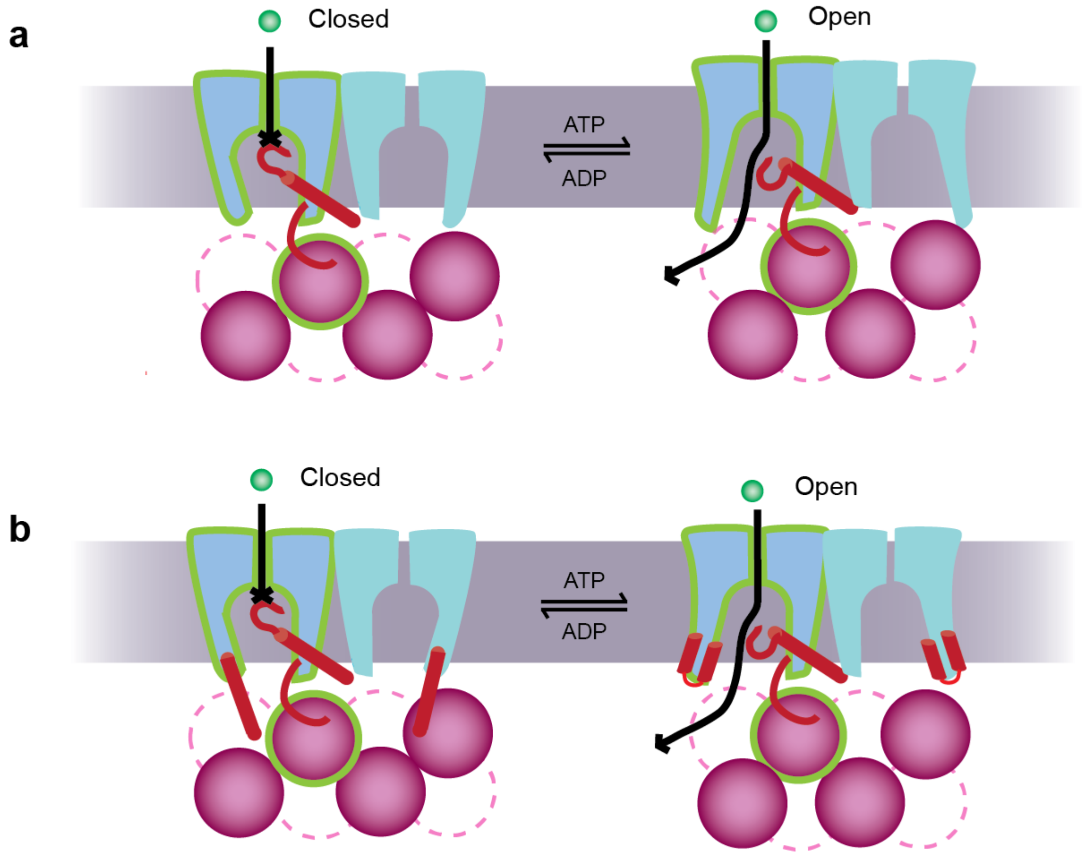

Supplementary Figure 11. Two proposed gating mechanisms in KtrB-KtrA

a. Gating mechanism of KtrB-KtrA proposed by Morais Cabral *et al.*[2,7]. Left, in the presence of ADP, KtrA forms an elongated gating ring, and the D1-D2 loop from KtrB does not interact with KtrA. Right, in the presence of ATP, KtrA contracts into a square shape, and interacts with the D1-D2 loop on KtrB to open the channel. **b.** Gating mechanism of KtrB-KtrA proposed by Hanelt *et al.*[8]. Left, in the presence of ADP, KtrA forms an elongated gating ring. The D1-D2 loop from KtrB converts into an extension of helix D1M2 and interacts with KtrA to keep the channel closed. Right, in the presence of ATP, KtrA contracts into a square shaped ring. The D1-D2 loop moves away from KtrB to open the channel.

Supplementary References:

1. Cao, Y. *et al.* Gating of the TrkH ion channel by its associated RCK protein TrkA. *Nature* **496**, 317–322 (2013).
2. Vieira-Pires, R. S., Szollosi, A. & Morais-Cabral, J. H. The structure of the KtrAB potassium transporter. *Nature* **496**, 323–328 (2013).
3. Huang, C. S., Pedersen, B. P. & Stokes, D. L. Crystal structure of the potassium-importing KdpFABC membrane complex. *Nature* **323–328**, 681–685 (2017).
4. Hryc, C. F. *et al.* Accurate model annotation of a near-atomic resolution cryo-EM map. *Proc. Natl. Acad. Sci.* **114**, 3103–3108 (2017).
5. Swint-Kruse, L. & Brown, C. S. Resmap: Automated representation of macromolecular interfaces as two-dimensional networks. *Bioinformatics* **21**, 3327–8 (2005).
6. Laskowski, R. A. & Swindells, M. B. LigPlot$^+$: Multiple ligand-protein interaction diagrams for drug discovery. *J. Chem. Inf. Model.* **51**, 2778–86 (2011).
7. Szollosi, A., Vieira-Pires, R. S., Teixeira-Duarte, C. M., Rocha, R. & Morais-Cabral, J. H. Dissecting the Molecular Mechanism of Nucleotide-Dependent Activation of the KtrAB K^+ Transporter. *PLoS Biol.* **14**, (2016).
8. Diskowski, M. *et al.* Helical jackknives control the gates of the double-pore K^+ uptake system KtrAB. *Elife* **6**, (2017).

Supplementary Table 1

Table 1 Data collection and refinement statistics (molecular replacement)

	TrkH-TrkA-ADP	TrkH-TrkA-ATPγS
**Data collection**		
Space group	C2	$P4_2$
Cell dimensions		
a, *b*, *c* (Å)	262.10 188.50 187.91	164.27 164.27 123.39
α, β, γ (°)	90.00 133.16 90.01	90 90 90
Resolution (Å)	3.53	3.80
R_{merge}	0.065(1.70)	0.073(1.56)
$I / \sigma I$	30.63(1.0)	38.90(1.9)
Completeness (%)	99.3 (98.1)	99.8(100)
Redundancy	6.8(6.2)	13.4(11.3)
**Refinement**		
Resolution (Å)	49.82 - 3.53	49.33 - 3.8
No. reflections	68,896	31,442
R_{work} / R_{free}	0.25/0.29	0.27/0.33
No. atoms	28,234	14,347
Protein	28,126	14,223
Ligand/ion	108	124
Water		
B-factors	80.37	39.75
Protein	80.32	39.52
Ligand/ion	94.04	66.36
Water		
R.m.s. deviations		
Bond lengths (Å)	0.005	0.05
Bond angles (°)	1.01	0.96
Ramachandran plot		
Favored (%)	94.90	93.46
Allowed (%)	4.66	6.22
Disallowed (%)	0.44	0.33

Supplementary Table 2
Cryo-EM data collection, refinement and validation statistics

	**TrkH-TrkA-ATP**
**Data collection and processing**	
Magnification	30,000
Voltage (kV)	300
Electron exposure (e–/Å^2)	80
Defocus range (μm)	0.5-3.5
Pixel size (Å)	1.23
Symmetry imposed	no
Initial particle images (no.)	
Final particle images (no.)	72,317
Map resolution (Å)	2.97
FSC threshold	0.142
Map resolution range (Å)	270-2.97
**Refinement**	
Initial model used (PDB code)	de novo initial model
Model resolution (Å)	low-passed to 60
FSC threshold	0.143
Model resolution range (Å)	3-60
Map sharpening *B* factor (Å^2)	-91
Model composition	
Non-hydrogen atoms	
Protein residues	20703
Ligands	0
B factors (Å^2)	51.49
Protein	51.49
Ligand	
R.m.s. deviations	
Bond lengths (Å)	0.01
Bond angles (°)	1.79
Validation	
MolProbity score	2.60
Clashscore	17.7
Poor rotamers (%)	5.69
Ramachandran plot	
Favored (%)	95.71
Allowed (%)	3.91
Disallowed (%)	0.38
