# Supplementary material for: TrkA undergoes a tetramer-to-dimer conversion to open TrkH which enables changes in membrane potential
Source: Nat Commun. 2020 Jan 28;11:547. doi: 10.1038/s41467-019-14240-9 (PMC6987127; doi:10.1038/s41467-019-14240-9)
Supplement: Supplementary file 4 — Description of Additional Supplementary Files [file 41467_2019_14240_MOESM4_ESM.pdf]

**Description of Additional Supplementary Files**

**File name:** Supplementary Movie 1

**Description:** Morph of TrkHA from the ADP bound to ATP bound state. This movie shows a morph from TrkHA-ADP to TrkHA-ATP. The HN1, N1-N1, and N2-N2 interfaces are shown sequentially in zoomed in views. The morph was produced in the program UCSF Chimera.
